# Supplementary material for: Evaluation of Marker Materials and Spectroscopic Methods for Tracer-Based Sorting of Plastic Wastes
Source: Polymers (Basel). 2022 Jul 29;14(15):3074. doi: 10.3390/polym14153074 (PMC9370613; doi:10.3390/polym14153074)
Supplement: Supplementary file 1 [file polymers-14-03074-s001.zip › Supplementary Material S1.pdf]

# Evaluation of Marker Materials and Spectroscopic Methods for Tracer-Based Sorting of Plastic Wastes

## Supplementary Material S1

**Christoph Olscher [1], Aleksander Jandric [1,*], Christian Zafiu [1] and Florian Part [1,2]**

1 Department of Water-Atmosphere-Environment, Institute of Waste Management, University of Natural Resources and Life Sciences, Muthgasse 107, 1190 Vienna, Austria; christoph.olscher@boku.ac.at (C.O.); christian.zafiu@boku.ac.at (C.Z.); florian.part@boku.ac.at (F.P.)
2 Bundesanstalt für Materialforschung und -Prüfung (BAM), 3.1 Fachbereich Gefahrgutverpackungen,
Unter den Eichen, 44-4612203 Berlin, Germany
* Correspondence: aleksander.jandric@boku.ac.at (A.J.)

**Table S1.** Terms used for the research on possible tracer substances/compounds in the search engines, divided into three categories: unspecific, substance specific, method specific.

| Search terms | | |
|---|---|---|
| **Unspecific** | **Substance specific** | **Method specific** |
| Tracer based sorting | Perylene tetraesters | XRF |
| Marker | Perylene diimides | NIR |
| Advanced materials | Quinacridone | UV-VIS |
| Excitation | Polyoxy-methylene (POM) | IR |
| Emission | Organic | LIBS |
| Luminescence | Inorganic | FTIR |
| Materials | Single walled carbon nano tubes | RAMAN |
| Nanocrystals | Quantum dots | Up-conversion fluorescence spectroscopy |

**Table S2.** Compatibility of POM with other polymers. This information is taken from VDI 2343 Blatt 5 - Recycling of electrical and electronic equipment - Material and thermal recycling and removal. M… mixable, I…immiscible, B…bonding agent required.

| | **ABS** | **ASA** | **PA** | **PBT** | **SB** | **PC** | **PE-LD** | **PE-HD** | **PES** | **PET** | **PMMA** | **POM** | **PP-H** | **PP-HI** | **PS** | **PVC** | **SAN** |
|---|---|---|---|---|---|---|---|---|---|---|---|---|---|---|---|---|---|
| **ABS** | - | M | B | I | I | M | I | I | I | I | M | I | I | I | I | M | M |
| **ASA** | M | - | B | I | I | M | I | I | I | I | M | I | I | I | I | M | M |
| **PA** | B | B | - | I | B | B | B | B | I | I | I | I | B | B | B | I | B |
| **PBT** | I | I | I | - | I | M | B | B | I | M | I | I | B | B | I | I | I |
| **SB** | I | I | B | I | - | I | B | B | I | I | I | I | B | B | M | I | I |
| **PC** | M | M | B | M | I | - | I | I | M | M | M | I | I | I | I | I | M |
| **PE-LD** | I | I | B | B | B | I | - | M | I | B | I | I | I | M | B | I | I |
| **PE-HD** | I | I | B | B | B | I | M | - | I | B | I | I | I | M | B | I | I |
| **PES** | I | I | I | I | I | M | I | I | - | I | I | I | I | I | I | I | I |
| **PET** | I | I | I | M | I | M | B | B | I | - | I | I | B | B | I | I | I |
| **PMMA** | M | M | I | I | I | M | I | I | I | I | - | I | I | I | I | M | M |
| **POM** | I | I | I | I | I | I | I | I | I | I | I | - | I | I | I | I | I |
| **PP-H** | I | I | B | B | B | I | I | I | I | B | I | I | - | M | B | I | I |
| **PP-HI** | I | I | B | B | B | I | M | M | I | B | I | I | M | - | B | B | B |
| **PS** | I | I | B | I | M | I | B | B | I | I | I | I | B | B | - | I | I |
| **PVC** | M | M | I | I | I | I | I | I | I | I | M | I | I | I | I | - | M |
| **SAN** | M | M | B | I | I | M | I | I | I | I | M | I | I | I | I | M | - |

**Table S3.** Examples of applications for POM polymers.

| Application | Components | Desired characteristics |
|---|---|---|
| Electrical and Electronic Equipment | Gear wheels, mechanical reinforces, clips, and other precision (durable) parts in printers, telephones, radio, television, washing machines, refrigerators and electric shavers | High mechanical stress resilience combined with high precision production and moulding |
| Automotive | Seat belt adjuster, speaker grille, door lock, automotive clips, air filtration system, fuel caps, fuel pump modules | High durability, low-gloss surface and other specific aesthetic characteristics, resistance to cleaning solutions and dimensional stability |
| Furniture | Fittings, locks, handles, hinges, curtain and door rollers, chairs, desk surfaces | High durability and mechanical stress resilience, resistance to cleaning solutions and water |
| Construction and buildings | Sleeves for point supports, valve components, door and window frames | Excellent combination of hardness, rigidity, and toughness, broad temperature range, water resistance |
| Household utensils and textiles | Spice mills, grinders, zip fasteners, clothing clips, vapour and e-cigarette accessories, knife handles, coffee brewers | High mechanical stress resilience combined with high precision production and moulding |
